# Knowledge and use of prognostic scales by oncologists and palliative care physicians in adult patients with advanced cancer: A national survey (ONCOPRONO study)

**DOI:** 10.1002/cam4.4467

**Published:** 2021-12-24

**Authors:** Raphaëlle Dantigny, Fiona Ecarnot, Guillaume Economos, Elise Perceau‐Chambard, Stéphane Sanchez, Cécile Barbaret

**Affiliations:** ^1^ Department of Supportive and Palliative Care Centre Hospitalier Universitaire Grenoble Alpes Boulevard de la Chantourne La Tronche France; ^2^ Department of Cardiology University Hospital Besançon Besançon France; ^3^ University of Burgundy Franche‐Comté Besançon France; ^4^ Department of palliative and supportive care Centre Hospitalier Universitaire de Lyon Sud Hospital Pierre Bénite France; ^5^ Department of Public Health and Performance Hôpitaux Champagne Sud Troyes France; ^6^ Laboratoire ThEMAS (Techniques pour l’évaluation et la Modélisation des Actions de Santé (TIMC‐IMAG: Technique de l’Ingénierie Médicale et de la Complexité‐Informatique, Mathématiques et Applications)) La Tronche France

**Keywords:** advanced cancer, national survey, palliative care, prognostic factors, prognostic scales

## Abstract

**Background:**

Prognostic scales exist to estimate patient survival in advanced cancer. However, there are no studies evaluating their use and practice. The objective of this study was to evaluate in a nationwide study the proportion of oncologists and palliative care physicians who had knowledge of these scales.

**Methods:**

A descriptive, national, cross‐sectional study was conducted via an online questionnaire to oncologists and palliative care physicians across France.

**Results:**

Palliative care physicians had better knowledge of the scales than oncologists (42.3% (*n* = 74) vs. 27.8% (*n* = 33), *p* = 0.015). The Palliative Performance Status (PPS) and Pronopall Scale were the best‐known (51.4% (*n* = 55) and 65.4% (*n* = 70), respectively) and the most widely used (35% (*n* = 28) and 60% (*n* = 48), respectively). Improved training in the use of these scales was requested by 85.4% (*n* = 251) of participants, while 72.8% (*n* = 214) reported that they did not use them at all. Limited training and lack of consensus on which scale to use were cited as the main obstacles to use.

**Conclusion:**

This is the first national study on the use of prognostic scales in advanced cancer. Our findings highlight a need to improve training in these scales and to reach a consensus on scale selection.

## Graphical abstract

The objective of this study was to nationally evaluate the proportion of oncologists and palliative care physicians who had knowledge of these scales. A descriptive national cross‐sectional study was conducted as an online questionnaire to oncologists and palliative care physicians across France. This is the first national study on the use of prognostic scales in advanced cancer and our findings highlights a need to improve training on such scales and to define a consensus on scale selection.

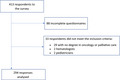

## 1 INTRODUCTION

Research has shown a negative impact of so‐called “aggressive” care on the quality of life (QOL) of cancer patients at the advanced palliative phase.^1^, ^2^, ^3^ Earle et al. developed a set of quality indicators to assess the intensity of end‐of‐life care.^4^ However, to date, routine practice remains far from optimal in this regard. Chemotherapy near the end‐of‐life remains frequent, with 10% to 40% of cancer patients receiving a new chemotherapy line within 1 month of death.^5^, ^6^, ^7^, ^8^, ^9^ There is a compelling need to align practices with recommendations, through improved anticipation of the terminal phase, and de‐escalation of the intensity of care as end‐of‐life approaches. To do this, a physician must be able to estimate how close the patient is to death, although it has been reported that oncologists tend to be overly optimistic in their estimations of life expectancy,^10^ over‐estimating survival on average by a factor of 5.3.

To enable improved estimation of life expectancy in patients with cancer, several prognostic scores exist,^11^, ^12^, ^13^, ^14^, ^15^, ^16^, ^17^, ^18^ and their use is widely established.^18^, ^19^, ^20^ Initially and primarily reserved for end‐of‐life patients,^11^, ^21^ some scores are now routinely used in patients still receiving curative therapy,^15^, ^22^ or at the early stages of palliative care.^22^ Yet, despite this proliferation of scores in recent years and the inclusion of some of them in national guidelines for supportive care in cancer,^23^, ^24^, ^25^ none of the key professional associations have accepted the complete integration of prognostic scores. Moreover, there is currently no international consensus regarding their role in patient management, or regarding which (if any) of the instruments should be recommended for use over another.

The aim of this study was to therefore describe the state of knowledge and the rate of use in routine practice of prognostic scores in advanced cancer. The primary objective was to evaluate the proportion of respondents who were aware of prognostic scales within their practice. The secondary objectives were: (i) to determine the proportion of respondents who use prognostic scores in their routine practice (frequency, circumstances, clinical utility); (ii) to determine the most widely known and frequently used scale(s); (iii) to investigate a potential relationship between the criteria included in the scores and the criteria applied by the respondents in their clinical practice to estimate life expectancy in patients with advanced cancer; and (iv) to assess respondents’ levels of interest in prognostic scales.

## 2 METHODS

A descriptive, cross‐sectional, national survey was conducted and was available online for a period of 6 weeks from June 01 to July 12, 2020.

### 2.1 Study population

Respondents meeting all the following criteria were eligible to participate in the study: (i) Physicians registered with the national medical council, (ii) working in the field of oncology or palliative care, and (iii) holders of the appropriate nationally recognized qualifications in oncology or palliative care.

We excluded participants that meet the following noninclusion criteria: (i) respondents who were qualified in neither oncology nor palliative care, (ii) physicians working in pediatric oncology, and (iii) physicians working in hematology.

According to data provided by the national medical council, the total number of registered physicians meeting the inclusion criteria were 5112, of which 4492 were registered oncologists (medical oncologists and physicians from other specialties with qualifications enabling them to practice oncology within their specialty area), and 682 physicians registered in palliative care.

### 2.2 Study design and implementation

A link to an online questionnaire (LimeSurvey) was sent to all physicians who were registered specialists in either oncology or palliative care in France. The questionnaire was available online for completion from June 01 to July 12, 2020. Physicians were contacted through regional cancer care networks (*N* = 12), and through the mailing list of the French Society for Palliative Care (SFAP).

### 2.3 Data recorded in the questionnaire

The text of the questionnaire is provided in the Supplementary Material. The survey was sectioned into six parts. The first section collected sociodemographic data. The second section investigated the respondents’ knowledge of the five prognostic scales (Palliative Performance Status (PPS), Palliative Prognostic (PaP) Score, Palliative Prognostic Index (PPI), Glasgow Prognostic Score (GPS), and the Barbot score or Pronopall score). The level of knowledge was assessed on a three‐level Likert scale, namely: (i) no knowledge (never heard of any of the instruments), (ii) limited knowledge (awareness that one or more of the scales exist, knows the names of one or more of the scales, knows some of the items included), and (iii) good knowledge (in‐depth knowledge of at least one of the scales and its component criteria). Participants were also asked to indicate which sources they used to obtain information on the scales or about training about palliative care (such as guidelines and professional associations). Section three enquired about the respondents’ use of prognostic scales. For participants that answered “yes” to its use, they were asked to indicate the frequency of use on each scale that was declared (rarely, often, frequently, daily), and the context in which they used it (choice from among 14 proposals compiled from a review of literature ‐ see Supplementary Material).

Participants were asked to indicate whether the prognostic scales were beneficial to them in their daily practice (never, sometimes, often, and always). Participants were also asked to indicate which scale they used most frequently. For self‐declared nonusers, the obstacles to the use of scales were recorded (choice among 11 proposals, see Supplementary Material).

The fourth section enquired about the clinical relevance of the criteria in the prognostic scales. The aim of this section was to investigate whether the criteria cited by the physicians overlapped with those of the prognostic scales. A list of 12 criteria were provided including clinical, biological, social, and psychological characteristics and respondents were asked which ones they applied in their daily practice to estimate life expectancy. Some of the criteria proposed as response modalities were also present in the prognostic scores, while some were factors reported in literature to be determinant in decision‐making among patients with advanced cancer.^24^

The last section of the questionnaire assessed the participants’ interest in prognostic scales. Participants were asked to respond using a 4‐point Likert scale (strongly agree, agree, disagree, and strongly disagree) regarding: (i) whether the evaluation of patient prognosis was important; (ii) whether the prognostic scales were useful for the purpose; (iii) whether they would like to learn more about the scales after having participated in our study; and (iv) whether they would like to use these scales in their daily medical practice.

### 2.4 Prognostic scales included in the study

A review of literature was performed via Medline and based on a recent review published in 2017.^18^ A large number of validated prognostic tools exist in the international literature. The choice of tools to include in this study was made based on the following criteria: (1) we retained any scale for which a validated French adaptation is available; and (2) among the seven main scales identified by a recent review of the literature, two were validated in only one study each and were therefore not retained for inclusion in this study (internal and external validity). Finally, five main prognostic scales were retained namely:

1. *PPS*,^11^, ^26^, ^27^, ^28^, ^29^, ^30^, ^31^ validated in patients with or without cancer followed up in palliative care, comprising six subjective criteria: physical performance (%) based on five observable parameters: the degree of ambulation, ability to do activities/extent of disease, ability to do self‐care, food/fluid intake, and state of consciousness
2. *PaP score*,^12^, ^20^, ^32^, ^33^, ^34^, ^35^ validated in patients with solid tumors not eligible for specific treatment (except palliative radiotherapy and hormone therapy), a mixed scale comprising both biological and clinical items, covering six criteria: dyspnea, anorexia, Karnofsky Performance Status (KPS), Clinical Prediction of Survival (CPS), total white blood count (WBC), and lymphocyte percentage
3. *PPI*,^13^, ^36^, ^37^, ^38^, ^39^, ^40^, ^41^, ^42^ validated in patients with cancer at the advanced palliative phase, comprising of five purely clinical criteria: Performance Status (PS), oral intake, edema, dyspnea at rest, and delirium.
4. *GPS*,^14^, ^22^, ^43^, ^44^ validated in patients with cancer (operable or not, patients receiving chemotherapy and/or radiotherapy), comprising two biological criteria: albumin and C‐reactive protein.
5. The *Barbot (or Pronopall) score*,^15^, ^45^, ^46^ validated in patients with cancer at the advanced palliative phase with an estimated survival of <6 months but excluding patients with hematological malignancies. This is a mixed score comprising four criteria: PS, number of metastatic sites, serum albumin, and lactate dehydrogenase.

### 2.5 Evaluation of the validity of our study questionnaire

The questionnaire was designed jointly by one palliative care physician and one oncologist, both qualified in research methodology. In addition, a literature review was performed to inform the question design and the areas to investigate. A methodologist then reviewed the questionnaire to ensure that the data could be properly collected, extracted, and analyzed. Finally, the questionnaire was piloted on seven physicians (a mix of oncologists and palliative care physicians) to verify comprehension, fluidity, and time required for completion.

### 2.6 Ethical considerations

Since this study was considered as an evaluation of professional practices, and in accordance with French legislation, Ethics Committee approval was not required for this study. However, the study was registered with the relevant national authorities (National Institute for Healthcare Data, INDS, under the number MR3512280520). Participants were informed that their participation was voluntary, and together with the questionnaire, participants also received an information leaflet, and a form allowing them to withdraw their consent. The data were anonymous, and a pseudonymization code was used to enable back‐identification of individual questionnaires via a personal code created by each participant at the end of the questionnaire.

### 2.7 Statistical analysis

Data were extracted from the online platform and analyses were performed using SPSS version 21.0 (IBM Inc). Quantitative data are presented as mean ± standard deviation (SD) or median [interquartile] and were compared using the Student *t* or Mann–Whitney *U* test as appropriate. Qualitative variables are presented as number (percentage) and were compared using the chi square or Fisher's exact test.

## 3 RESULTS

**FIGURE 1:**
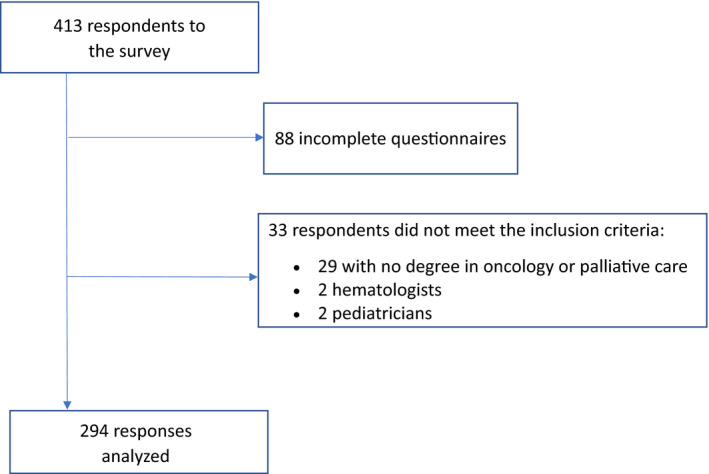
Flow chart for the ONCOPRONO study, a national survey to assess the knowledge and use of prognostic scales by oncologists and palliative care physicians in adults with advanced cancer

After the exclusion of incomplete questionnaires (88 partial answers among 413 respondents), the participation rate was 9.6% (325 responses from a total of 3408 contacted). The participation rate among palliative care physicians was higher compared to oncologists at 38% (175 responses out of 454 contacted), whereas only 4% of oncologists participated in the survey (119 responses out of 2954 contacted). A total of 294 complete responses were analyzed (Figure 1). The characteristics the respondents are detailed in Table 1.

**TABLE 1:** Demographic and professional characteristics of respondents from the ONCOPRONO study

Demographical and professional characteristics of respondents	All (*N* = 294)	Oncologists (*N* = 119)	Palliative care physicians (*N* = 175)
Age (years, SD)	43.8 (10.5)	41.8 (10.2)	44.7 (11.4)
Sex			
Female	170 (57.8%)	62 (52.1%)	108 (61.7%)
Male	124 (42.2%)	57 (47.9%)	67 (38.3%)
Type of establishment			
Academic teaching hospital	80 (27.2%)	34 (28.6%)	46 (26.3%)
Cancer center	54 (18.4%)	36 (30.2%)	18 (10.3%)
Public (general, non‐academic) hospital	112 (38.1%)	35 (29.4%)	77 (44.0%)
Private hospital	48 (16.3%)	14 (11.8%)	34 (19.4%)
Medical specialty			
Oncology (medical and radiotherapy)	80 (27.2%)	73 (61.3%)	7 (4.0%)
Medical specialty except oncology	209 (71.1%)	44 (37.0%)	165 (94.3%)
Family practice	159 (54.1%)	9 (7.6%)	150 (85.7%)
Other medical specialty	50 (17.0%)	35 (29.4%)	15 (8.6%)
Surgery	2 (0.7%)	2 (1.7%)	0 (0%)
Experience			
≤5 years	80 (27.2%)	30 (25.2%)	50 (28.6%)
6–10 years	82 (27.9%)	33 (27.7%)	49 (28%)
11–20 years	83 (28.2%)	34 (28.6%)	49 (28%)
≥20 years	49 (16.7%)	22 (18.5%)	27 (15.4%)
Frequency of collaboration			
Never to rarely	—	10 (7.7%)	9 (4.7%)
Often to frequently	—	120 (92.3%)	179 (95.3%)

Among the 294 participants, 10.2% (*n* = 30) were not aware of the existence of prognostic scales, and 53.4% (*n* = 157) reported limited knowledge (existence/name some criteria). Palliative care physicians were more knowledgeable about prognostic scales than oncologists (42.3% (*n* = 74) vs. 27.8% (*n* = 33) respectively, *p* = 0.015).

Overall, 27.2% of participants (*n* = 80) reported using the prognostic scales. There was no difference between palliative care physicians and oncologists in terms of use (30.3% (*n* = 53) vs. 22.7% (*n* = 27) respectively, *p* = 0.15). Table 2 presents how well each scale was known and how widely it was used by both groups of physicians. The Pronopall score was both the best known (65.4%, *n* = 70) and most widely used (60%, *n* = 48) followed by the PPS scale, both in terms of knowledge (51.4%, *n* = 55) and use (35%, *n* = 28). Both knowledge (68.9%, *n* = 51 vs. 12.1%, *n* = 4, *p* < 0.001) and use (52.8%, *n* = 28 vs. 0%, *p* < 0.001) of the PPS scale were greater among palliative care physicians than among oncologists, whereas there was no difference between the two specialities regarding the Pronopall score.

**TABLE 2:** The most widely known and used prognostic scales, as reported by respondents in the ONCOPRONO study

Prognostic scales: most known and used	Those with good knowledge (*N* = 107)	Oncologists with good knowledge (*N* = 33)	Palliative care physicians with good knowledge (*N* = 74)	*p*‐value	All users (*N* = 80)	Those using the scales and working in oncology (*N* = 27)	Those using the scales and working in palliative care (*N* = 53)	*p*‐value
Palliative Performance Scale (PPS)^11^	55 (51.4%)	4 (12.1%)	51 (68.9%)	<0.001	28 (35%)	0 (0%)	28 (52.8%)	<0.001
Palliative Prognostic Score (PaP)^12^	18 (16.8%)	2 6.1%)	16 (21.6%)	0.04	3 (3.7%)	0 (0%)	3 (5.7%)	0.52
Barbot score (Pronopall score)^13^	70 (65.4%)	21 (63.7%)	49 (66.2%)	0.79	48 (60%)	20 (74%)	28 (52.8%)	0.11
Palliative Prognostic Index (PPI)^14^	18 (16.8%)	1 (3.0%)	17 (23%)	0.01	4 (5%)	1 (3.7%)	3 (5.7%)	1.00
Glasgow Prognostic Score (GPS)^15^	12 (11.2%)	3 (9.1%)	9 (12.2%)	0.64	3 (3.7%)	1 (3.7%)	2 (3.8%)	1.00

The rate of use of the scales varied, with most respondents stating they used them between several times per month (38.75%, *n* = 31) and several times per week (28.75%, *n* = 23). The most common circumstances in which the scales were used are described in Table 3. Overall, the main reason for their use was with a view to limiting chemotherapy (*n* = 44, 55.7%). For oncologists, the scales were used primarily to limit chemotherapy (84.6%, *n* = 22), limit targeted therapies (42.3%, *n* = 11), and during discussions prior to admission to intensive care (30.8%, *n* = 8).

**TABLE 3:** The three most important situations for the use of prognostic scales, among self‐reported scale users in the ONCOPRONO study

The three most important situations for use of prognostic scales, among users		All users (*N* = 79)	Users working in oncology (*N* = 26)	Users working in palliative care (*N* = 53)	*p*‐value
Oncologists	Limitation of chemotherapy	44 (55.7%)	22 (84.6%)	22 (41.5%)	<0.001
	Limitation of targeted therapies including immunotherapy	14 (17.7%)	11 (42.3%)	3 (5.7%)	<0.001
	Discussion before going to the intensive care unit	22 (27.8%)	8 (30.8%)	14 (26.4%)	0.68
Palliative care physicians	Limitation of invasive procedures (drain, gastrostomy, and palliative surgery)	31 (39.2%)	6 (23.1%)	25 (47.2%)	0.06
	Limitation of artificial nutrition	30 (38%)	6 (23.1%)	24 (45.3%)	0.06
	Drafting of care limitations/precisions regarding intensity of care	29 (36.7%)	6 (23.1%)	23 (43.4%)	0.07

Palliative care physicians reported using the scales to limit invasive treatment(s) (47.2%, *n* = 25), limit artificial nutrition (45.3%, *n* = 24), and for the physicians to decide on the limitations of care (43.4%, *n* = 23). There was a difference between the rate of use between oncologists and palliative care physicians in terms of limitation of chemotherapy and targeted therapy. In total, among the 79 physicians who reported using the scales, 98.75% thought that they were clinically relevant, with 52.5% (*n* = 42) stating that they sometimes found them helpful, 36.25% (*n* = 29) often, and 10% (*n* = 8) always.

The main obstacles to wider use of the prognostic scales as cited by the respondents are detailed in Table 4. The main barriers cited were a lack of training (54.2%, *n* = 116), the lack of a consensus regarding scale selection (28.5%, *n* = 61), and the lack of any need for such scores, because experience was stated to be more useful than using the scores (26.2%, *n* = 56).

**TABLE 4:** Obstacles to the use of prognostic scales cited by self‐reported nonusers of the scales in the ONCOPRONO study

Obstacles to use of prognostic scales	All non‐users (*N* = 214)	Non‐users working in oncology (*N* = 92)	Non‐users working in palliative care (*N* = 122)	*p* value
Scales not recognized	46 (21.5%)	25 (27.2%)	21 (17.2%)	
Too long	18 (8.4%)	11 (11.9%)	7 (5.7%)	
Too many scales in oncology	37 (17.3%)	26 (28.3%)	11 (9.0%)	<0.001
Too difficult to implement	11 (5.1%)	5 (5.4%)	6 (4.9%)	
Unreliable	11 (5.1%)	2 (2.2%)	9 (7.4%)	
Useless	56 (26.2%)	23 (25%)	33 (27.0%)	0.73
No consensus on which one to use	61 (28.5%)	28 (30.4%)	33 (27.0%)	0.58
Sometimes invasive (blood test)	14 (6.5%)	0 (0%)	14 (11.5%)	
No training in use of scales	116 (54.2%)	57 (62%)	59 (48.3%)	0.04
If palliative care practitioners do not use them, it would seem they are not useful	19 (8.9%)	14 (15.2%)	5 (4.1%)	

Most respondents (96.3%, *n* = 283) searched for guidelines on supportive and palliative care one or several times per year (39.8%, *n* = 117), one or several times per month (47%, *n* = 138), or even one to several times per week (9.5%, *n* = 28). The preferred sources of information in France were national professional associations such as the French‐language Society for Supportive and Palliative Care (AFSOS) (73.5%, *n* = 208), and the French Society for Palliative Care (SFAP) (70.7%, *n* = 200), followed by publications in the scientific literature (61.8%, *n* = 175), presentations at congresses (48.8%, *n* = 138) and international professional associations such as the European Society for Medical Oncology (ESMO) (24.7%, *n* = 70) and the American Society of Clinical Oncology (ASCO) (18.4%, *n* = 52).

The three most cited criteria used by the physicians to evaluate prognosis in their patients were general status (*n* = 249, 84.7%), findings on clinical examination (such as delirium, dyspnea, and edema) (*n* = 157, 53.4%) and clinical undernutrition (*n* = 114, 38.8%) (Table 5). Overall, 84.4% (*n* = 248) agreed that the evaluation of prognosis in this population was important, and 69.8% (*n* = 205) agreed that the prognostic scores were useful for assessing prognosis, while 85.4% (*n* = 251) expressed a desire to receive training in their use, and 70.4% (*n* = 207) reported that they would like to use the scales in practice.

**TABLE 5:** The three most important prognostic criteria for assessing patient prognosis as reported by respondents in the ONCOPRONO study

Prognostic criteria to assess patient prognosis (the three most important)	All respondents (*N* = 294)	Respondents working in oncology (*N* = 119)	Respondents working in palliative care (*N* = 175)	*p*‐value
Clinical undernutrition	114 (38.8%)	45 (37.8%)	69 (39.4%)	0.69
General status (Performance Status or Karnofsky Index)	249 (84.7%)	106 (89.1%)	143 (81.7%)	0.08
Clinical Prediction of Survival	70 (23.8%)	32 (26.9%)	38 (21.7%)	0.31
Patient age	9 (3.1%)	8 (6.7%)	1 (0.6%)	0.002
Patient psychology	17 (5.8%)	3 (2.5%)	14 (8.0%)	0.04
Response to previous chemotherapy	66 (22.4%)	43 (36.1%)	23 (13.1%)	<0.001
Number of metastatic sites	26 (8.8%)	3 (2.5%)	23 (13.1%)	<0.01
Location of primary cancer	18 (6.1%)	13 (10.9%)	5 (2.8%)	<0.01
Patient comorbidities	75 (25.5%)	33 (27.7%)	42 (24.0%)	0.47
Social context	2 (0.7%)	1 (0.8%)	1 (0.6%)	1.00
Clinical criteria (including confusion, dyspnea, and edema)	157 (53.4%)	46 (38.6%)	111 (63.4%)	<0.01
Biological criteria (including Albumin, CRP, and LDH)	64 (21.8%)	17 (14.3%)	47 (26.8%)	<0.001

## 4 DISCUSSION

This is the first study to describe the state of knowledge and frequency of use of prognostic scales for the evaluation of expected survival in patients with advanced cancer among oncologists and palliative care physicians. A key finding of this study was that prognostic scales for assessing expected survival were still relatively poorly known among oncologists and palliative care physicians in France, with 63.6% of physicians indicating that they knew little about them, if anything at all.

The obstacles to the wider use of these scales are a need for training in their use/interpretation, and the lack of consensus about which scale to use. To address this need, physicians reported that they relied on national and international guidelines and other scientific publications in the international literature to further their knowledge. However, guidelines providing a consensus about the role of these scales in the discussions surrounding patient management are still lacking. Furthermore, the dissemination of knowledge in this specific field may be impeded by the fact that major therapeutic innovations generate greater interest and prognostic scales may not benefit from the same level of attention. Another explanation why these scales were not widely known could reside in the level of education provided in primary palliative care, which enables more recently graduated physicians in all disciplines to learn basic skills in palliative care, and ideally should prompt them to reflect in a more systematic way on the intensity and/or limitation of care for their patients. At present, the medical curriculum for future oncologists in France provides insufficient training in palliative care skills, with the result that many of them lack knowledge and experience.^47^, ^48^, ^49^

Another finding of this study was that the prognostic scales we asked about were not widely used, with only slightly more than a quarter of respondents (27.2%, (*n* = 80)) declaring that they used them. The low rate of use can naturally be a corollary of the fact that they were not widely known among our respondents, but may also be partially due to the constraints that their use involves. Indeed, such scales may be perceived as time‐consuming, requiring time for completion, and constituting an additional tool to be applied on top of evaluations for pain, nutrition, anxiety, mental issues, and social context in patients whose management is already particularly long and complex. Furthermore, some physicians may simply not feel the need; indeed, there seemed to be a nonnegligible proportion of physicians who believed that their clinical judgement and experience was more helpful in estimating prognosis.

Among the participants who reported knowing and using the scales, the Pronopall and PPS scales were the most widely known and used, whereas the PaP, PPI, and Glasgow Prognostic Score were less commonly employed. There are several potential explanations for this finding. First and foremost, the Pronopall and PPS studies are the only two that have been validated in the French language.^11^ The Pronopall study is not widely known internationally, with the seminal publication^45^ appearing after the review of the literature published in 2017.^18^ It was developed and validated by a French group,^45^, ^46^, ^50^ and is cited in the French guidelines for supportive and palliative care^23^ resulting in relatively wide dissemination within France. Furthermore, the Pronopall score is easy to implement (comprising four factors) and corresponds to a population of patients with advanced cancer in the palliative phase, evaluating survival at 2 months in these patients.

Regarding the PPS scale, it is also easy to use and is based solely on clinical criteria. It is suitable for use in a large population of palliative care patients, with or without cancer, and with solid tumors or hematological malignancies. The other tools investigated in this study (PaP, PPI, and Glasgow scale) were found to be less frequently used since they were not validated in the French language and the timing of evaluation using these scales is less relevant in view of the intended use. Indeed, the PaP scale estimates the probability of survival at 30 days while the PPI scale estimates the likelihood that patients will live longer than 3 to 6 weeks. These time frames are sometimes too late and too close to the time of death for any meaningful discussions about accompaniment at the end‐of‐life.

Regarding the Glasgow Prognostic Score, it can be used in a range of situations, however, the relevant study must be identified each time to extract the applicable survival rates which makes its use somewhat more cumbersome. Finally, the PPI and PaP scales both include a larger number of variables (five and six, respectively), and the PPI may be time‐consuming to administer, since one of its component parts is the estimation of the PPS score.

This study provides insights into the circumstances in which oncologists and palliative care physicians use prognostic tools for estimating survival in advanced cancer patients. Indeed, oncologists reported that they mainly used prognostic scales to justify limitation of specific treatments, whereas palliative care physicians stated that their use of these scales was above all to limit therapies when they started to become unreasonably superfluous (such as invasive procedures and artificial nutrition). This difference in the determination of the scores between the two medical specialties is likely related to the consistently pervasive dichotomy between oncologists and palliative care physicians. The ASCO guidelines^51^, ^52^, ^53^ validate and encourage the integration of palliative care into the healthcare pathway of patients as soon as they receive a diagnosis of metastatic cancer. Nevertheless, palliative care is still often initiated too late^54^, ^55^ and plays only a minor role in the discussions surrounding withdrawal of oncological therapies, despite its contribution in these cases.^56^

### 4.1 Perspectives

We investigated whether the criteria that oncologists and palliative care physicians spontaneously use to guide their clinical decisions were similar to those that are used in the various scores. The criteria most frequently cited by the respondents were the patient's general state, findings on clinical examination (delirium, dyspnea, edema (*n* = 157, 53.4%), and clinical undernutrition (*n* = 114, 38.8%). Looking at the scales considered in this study, general status could be seen as a constituent component of all of them (except the Glasgow score, which is based solely on biological variables) and the findings of the clinical examination and clinical undernutrition are included in three out of the five scales (PPS, PaP, and PPI). Yet, while there appears to be some overlap, there is no real consensus, since one quarter of respondents preferred to trust their own subjective evaluation, with 70 (23.8%) preferring a clinical estimate of survival, and only around one in five (*n* = 64, 21.8%) using objective biological criteria as a basis for decision‐making. Relying on the physician's subjective evaluation can be misleading, as has previously been demonstrated by Christakis et al.,^10^ who reported that oncologists tended to overestimate survival on average by a factor of 5.3.

Therefore, prognostic scales that are objective and reproducible could help to formalize a physician's clinical perception, rank the numerous patient‐ and disease‐related factors affecting survival, and retain only those that are most determinant in terms of prognosis. Prognostic scales should be seen as an aid to decision‐making within the larger framework of an objective self‐evaluation of the relevance of the decisions. Despite their value, prognostic scales have not yet become decisional algorithms that can fully replace human decision‐making. There was clearly a need among respondents to receive training in the use of these scores and implement them more widely in their practice. A first actionable item would be to stimulate debate at national level among oncologists and palliative care physicians, with a view to improving communication and education about prognostic scales. Our study could serve as an entry point into this debate. A second actionable item would be to hold a Delphi group at national or international level to achieve consensus and/or provide clear recommendations on the most appropriate, reliable, and user‐friendly scales, and the contexts in which they should be used in routine practice. There was also a clear educational rationale to include prognostic scales in official recommendations.

### 4.2 Study limitations

This study had some limitations. First, the main limitation was the low rate of participation, estimated at approximately 10% of all oncologists, and palliative care physicians in France. However, it should be noted that there was uncertainty surrounding the actual number of physicians who received the questionnaire (i.e., the denominator). As there is currently no national mailing list of all specialists in oncology and/or palliative care, the only means to disseminate the questionnaire was to use regional mailing lists, which may have varying degrees of exhaustiveness and preclude identification of the number and qualifications of the physicians targeted. The ongoing COVID‐19 pandemic likely affected response rates as well with some networks for disseminating information refusing to participate in the circulation of the questionnaire due to the already excessive workload of their physicians. Similarly, the questionnaire was initially planned for dissemination in April 2020, but due to global outbreak, the start date was postponed to June 2020 leaving only a short time‐window of 6 weeks for dissemination and collection of responses.

Lastly, the use of the questionnaire may have had potential for bias, notably an overestimation by the respondents of their rate of use through social desirability bias and an aversion to appearing less knowledgeable. However, in view of the low rates of respondents who declared that they knew and used them, this is unlikely to have affected the results substantially. There was also a potential for selection bias, although the questionnaire was distributed and responses were obtained in all 12 regions of the country, which pleads in favor of the representativeness of our data. Although the low rate of response may limit the interpretation and generalizability of the results, this remains the first study to address this question.

## 5 CONCLUSION

In this study, we found that the major prognostic scales for estimating life expectancy in advanced cancer patients were neither well known nor widely used in France. Oncologists and palliative care physicians both expressed a desire for better training in the use of such scales and would be receptive to recommendations with a consensus on scale selection. Prognostic scales for predicting life expectancy in patients with advanced cancer cannot be used as a stand‐alone instrument but should be considered as an additional tool in the armamentarium of physicians to inform their discussions with the patient, family, and other healthcare providers.

## CONFLICT OF INTEREST

The authors have no conflict of interest to declare.

## AUTHOR CONTRIBUTIONS

Study conception: Raphaelle Habert‐Dantigny and Cécile Barbaret. Study design: Raphaelle Habert‐Dantigny and Cécile Barbaret. Data acquisition: Raphaelle Habert‐Dantigny and Cécile Barbaret. Data analysis and interpretation: Stéphane Sanchez, Raphaelle Habert‐Dantigny, and Cécile Barbaret. Statistical analysis: Stéphane Sanchez, Raphaelle Habert‐Dantigny, and Cécile Barbaret. Manuscript preparation: Raphaelle Habert‐Dantigny, Cécile Barbaret, Fiona Ecarnot, Stéphane Sanchez, Guillaume Economos, and Elise Perceau‐Chambard.

## ETHICAL APPROVAL

Since this study was considered as an evaluation of professional practices, and in accordance with French legislation, Ethics Committee approval was not required for this study. However, the study was registered with the relevant national authorities (National Institute for Healthcare Data, INDS, under the number MR3512280520). Participants were informed that their participation was voluntary, and together with the questionnaire, participants also received an information leaflet, and a form allowing them to withdraw their consent.

## Supporting information

Supplementary Material

## Data Availability

Our data are only available upon request due to the proprietary rights and legal restrictions at the University Hospital of Grenoble, France, however, they can be made available by written request addressed to our administration office at: accueilrecherche@chu-grenoble.fr.
