# Supplementary material for: Knowledge and use of prognostic scales by oncologists and palliative care physicians in adult patients with advanced cancer: A national survey (ONCOPRONO study)
Source: Cancer Med. 2021 Dec 24;11(3):826–37. doi: 10.1002/cam4.4467 (PMC8817080; doi:10.1002/cam4.4467)
Supplement: Supplementary file 1 — Supplementary Material [file CAM4-11-826-s001.docx]

**Supplementary Material: Questionnaire of the ONCOPRONO study**

**Part I: Socio-demographic data:**

1. What region of France do you work in? (Please only select one answer):

Auvergne-Rhône-Alpes/Bourgogne-Franche-Comté/Bretagne/Centre-Val de Loire/Corse /Grand Est/Hauts-de-France/Île-de-France/Normandie/Nouvelle-Aquitaine/Occitanie/Pays de la Loire/Provence-Alpes-Côte d'Azur

1. Sex (Please only select one answer):

- Female
- Male

1. What is your age: ……..
2. In what type of environment do you work in:

- Private practice (Clinic or private practice)
- University teaching hospital
- Specialized Cancer Centre (Centre de Lutte Contre le Cancer, CLCC)
- Public (Non-academic) hospital
- Other (Please specify):

1. In which type of department(s) do you currently work in:

- Medical oncology department (hospitalisation/outpatients/consultation)
- Palliative care department (mobile palliative care team/palliative care ward)
- Other medical specialty
- Surgical specialty
- Home-hospitalization service
- Care provision network
- Other (Please specify):

1. Please indicate which of the following diplomas you hold:

- Medical oncology specialization
- Onco-radiotherapy specialization
- Oncology qualification
- Validation of experience in oncology
- Oncology specialization
- Palliative care specialization
- Inter-university diploma in palliative care
- university diploma in palliative care
- I have no diplomas in either oncology or palliative care
- Other (Please specify):

1. What was your initial training or specialty? (Please only select one answer):

- Family medicine
- Medical oncology
- Onco-radiotherapy
- Respiratory medicine
- Hepato-gastro-enterology
- Dermatology
- Medical specialty other than above (Please specify):
- Surgical specialty (Please specify):

1. How long have you been working in oncology/palliative care? (Please only select one answer):

- ≤5 years
- 6 to 10 years
- 11 to 20 years
- 21 to 30 years
- >30 years
- I work in both oncology and palliative care

1. If you work in oncology, how often do you work with a palliative care team? (Please only select one answer):

- Never
- Rarely: One to several times a year
- Often: One to several times a months
- Frequently: One to several times a week
- Not applicable

1. If you work in palliative care, how often are you contacted by an oncology team? (Please only select one answer):

- Never
- Rarely: One to several times a year
- Often: One to several times a months
- Frequently: One to several times a week
- Not applicable

**Part II: Evaluation of the level of knowledge of validated prognostic scales and sources of knowledge in palliative care**

1. Regarding prognostic scales in oncology in patients with advanced cancer, how would you rate your knowledge of these scales? (Please only select one answer):

- I do not know what these scales are
- I just know that these scales exist
- I have heard the name of some of these scales
- I know some of the criteria that are included in these scales
- I know at least one of these scales and its components

If in Q11, you answered “I don’t know what these scales are”, please go straight to Q14.

1. Among the prognostic scales in oncology, which one(s) do you recognize? (Please answer this question if you answered “I just know they exist/I’ve heard the name of some of these scales” to Q11) (please select all those that apply):

- Palliative Performance Scale (PPS)
- Palliative Prognostic Score (PaP)
- Barbot Score (score PRONOPALL)
- Palliative Prognostic Index (PPI)
- Glasgow Prognostic Score (GPS)
- None of the above

1. Among the prognostic scales in oncology, which one(s) are you familiar with (knowing the component criteria)? (Please answer this question if you answered “I know some of the criteria included in these scales/I know at least one of these scales and its components” to Q11) (please select all those that apply):

- Palliative Performance Scale (PPS)
- Palliative Prognostic Score (PaP)
- Barbot Score (score PRONOPALL)
- Palliative Prognostic Index (PPI)
- Glasgow Prognostic Score (GPS)
- None of the above

1. Do you ever search for recommendations/guidelines about palliative and supportive care? (Please only select one answer):

- Never
- Rarely: one to several times a year
- Often: one to several times a months
- Frequently: one to several times a week

1. What sources or types of document do you use to find information about good practice in palliative and/or supportive care? (Please answer this question if you answered “Rarely”, “Often” or “Frequently” to Q14) (Please select all those that apply):

- Guidelines from the Association Francophone des Soins Oncologiques de Support (AFSOS)
- Guidelines from the Société Française d’Accompagnement et de Soins Palliatifs (SFAP)
- ESMO guidelines
- ASCO guidelines
- HAS (French national health authority) guidelines
- At congresses
- In industry-sponsored meetings
- I read scientific articles and journals
- Other

**Part III: Evaluation of your use of prognostic scales**

1. Do you use one or more prognostic scales in routine practice, for patients with advanced cancer at the palliative phase (Please only select one answer):

- Yes
- No

If Yes, then please answer Q17 Q18 Q19 Q20 ± Q21

If No, then please go straight to Q22

1. Among the following prognostic scales in oncology, which one(s) do you use? (Please select all those that apply):

- Palliative Performance Scale (PPS)
- Palliative Prognostic Score (PaP)
- Barbot (or PRONOPALL) Score
- Palliative Prognostic Index (PPI)
- Glasgow Prognostic Score (GPS)
- Other : Please specify :

1. How often do you use them in routine practice? (Please only select one answer):

- Rarely, one to several times a year
- Often, one to several times a month
- Frequently, one to several times a week
- Daily, one to several times a day

1. When you use these scales, what situations do you use them for? (Please select all those that apply):

- Limitation of chemotherapy
- Limitation of targeted therapy including immunotherapy
- Limitation of antibiotic therapy
- Limitation of radiotherapy
- Limitation of artificial nutrition
- Discussion before admission to ICU or continuous care
- Drafting of care limitations or for providing indications about intensity of care, to inform colleagues who will be on duty or on call
- Limitation of cardiopulmonary resuscitation
- Presentation to a palliative care
- Criterion for admission to palliative care unit
- Limitation of invasive procedures (drain, gastrostomy and palliative surgery)
- To estimate life expectancy of a patient at the request of the family
- To estimate life expectancy of a patient at the request of the patient
- Other

1. In your experience, did the use of these scales help you in your choice? (Please only select one answer):

- Never
- Sometimes
- Often
- Always

If you answered “Sometimes”, “Often” or “Always” to Q20, please answer Q21:

1. If you found these scales helpful at any time, in which situation(s) were they most use? (Please select a maximum of 3 answers)

- Limitation of chemotherapy
- Limitation of targeted therapy including immunotherapy
- Limitation of antibiotic therapy
- Limitation of radiotherapy
- Limitation of artificial nutrition
- Discussion before admission to ICU or continuous care
- Drafting of care limitations or for providing indications about intensity of care, to inform colleagues who will be on duty or on call
- Limitation of cardiopulmonary resuscitation
- Presentation to a palliative care
- Criterion for admission to palliative care unit
- Limitation of invasive procedures (including drain, gastrostomy and palliative surgery)
- To estimate life expectancy of a patient at the request of the family
- To estimate life expectancy of a patient at the request of the patient
- Other

If you answered No to Q16, please answer Q22 and Q23:

1. If you don’t use prognostic scales, what are the obstacles preventing you from using them? (Please select all those that apply):

- I don’t know these scales; I didn’t know they existed
- They are too long to implement; I do not have the time
- There are too many scales in oncology, I cannot do them all
- They are too complex to use
- They are not reliable
- They are not useful. Experience is more helpful in evaluating patient prognosis
- There is no consensus regarding these scales, I don’t know which one to use
- They sometimes require invasive procedures (such as blood tests)
- I was never trained in their use
- Since our palliative care physicians do not use them, I assume they are not useful
- Other

1. In which situations do you encounter difficulties in medical decision-making, and where it would be helpful to you to be able to better evaluate patient prognosis (Please select all those that apply):

- Limitation of chemotherapy
- Limitation of targeted therapy including immunotherapy
- Limitation of antibiotic therapy
- Limitation of radiotherapy
- Limitation of artificial nutrition
- Discussion before admission to ICU or continuous care
- Drafting of care limitations or for providing indications about intensity of care, to inform colleagues who will be on duty or on call
- Limitation of cardiopulmonary resuscitation
- Presentation to a palliative care
- Criterion for admission to palliative care unit
- Limitation of invasive procedures (including drain, gastrostomy and palliative surgery)
- To estimate life expectancy of a patient at the request of the family
- To estimate life expectancy of a patient at the request of the patient
- Other
- None of these situations

1. In your work environment, are these scales used during multidisciplinary meetings when discussing patients with advanced cancer at the palliative phase: (Please select all those that apply) :

- Never
- Sometimes
- Often
- Always
- Only by the oncology specialists participating in the multidisciplinary meeting
- Only by the palliative care specialists participating in the multidisciplinary meeting
- I don’t know because I don’t attend multidisciplinary meetings

**Part IV: Utility of prognostic scales**

1. Among the following clinical criteria, which do you think are the most relevant for evaluating patient prognosis? (Please select a maximum of 2 answers)

- General status (Performance Status or Karnofsky index)
- Presence of delirium
- Age of the patient
- Presence of dyspnea
- Severe malnutrition
- Presence of oedema

1. Among the following biological criteria, which do you think are the most relevant for evaluating patient prognosis? (Please select a maximum of 2 answers)

- CRP level
- Albumin level
- Vitamin B12 level
- LDH level
- Leukocytes and/or lymphocytes
- Impaired renal function
- Impaired liver function

1. In practice, what prognostic criteria do you mainly use for your medical decisions about withholding or withdrawal of care (considering your responses about the situations where you use the scales, or the situations where you encounter difficulties)? (Please select a maximum of 3 answers)

- Clinical malnutrition
- General status (Performance Status or Karnofsky index)
- Clinical estimation of life expectancy based on experience
- Age of the patient
- Patient’s mental status
- Response to prior chemotherapy
- Number of metastatic sites
- Location of the primary tumour
- Patient’s comorbidities
- Patient’s social context
- Clinical criteria (dyspnea, delirium, œdema)
- Biological criteria (albumin, CRP, LDH, leukocytes)

1. In your clinical experience, what other indicator(s) could be used to improve the evaluation of patient prognosis?

(Free text answer)

1. Regarding the use of immunotherapy and new therapeutic options, as a prescriber or observer:

For each statement, please indicate if you strongly agree/agree/disagree/strongly disagree:

- You would be more inclined to give immunotherapy than chemotherapy to a patient the advanced palliative phase/You would more readily agree to immunotherapy rather than chemotherapy for a patient the advanced palliative phase.
- Currently available prognostic scores should be updated to take account of new therapies.
- In view of the progress in molecular biology and personalized medicine, new biological indicators could help to better evaluate patient prognosis.

**Part V: What did you think of this study?**

1. Regarding prognostic scales:

For each statement, please indicate if you strongly agree/agree/disagree/strongly disagree:

- In my opinion, the evaluation of patient prognosis is an important problem
- Prognostic scales seem to be an invaluable tool for estimating life expectancy in these patients
- This study has made me want to learn more about these scales
- This study has made me want to use these scales

**Part VI: Re-identifying your questionnaire responses**

1. To exercise your right to access, rectify or delete your responses, please answer the following questions. The answers will be used as a code to back-identify your questionnaire, while preserving your anonymity

- Mother’s initials:

- Father’s initials:

- Choose two letters from the alphabet:

- Choose two numbers (between 0 and 9):
